# Thickness-Dependent Dark-Bright Exciton Splitting and Phonon Bottleneck in CsPbBr_3_-Based Nanoplatelets Revealed via Magneto-Optical Spectroscopy

**DOI:** 10.1021/acs.nanolett.2c01826

**Published:** 2022-08-29

**Authors:** Shuli Wang, Mateusz Dyksik, Carola Lampe, Moritz Gramlich, Duncan K. Maude, Michał Baranowski, Alexander S. Urban, Paulina Plochocka, Alessandro Surrente

**Affiliations:** † Laboratoire National des Champs Magnétiques Intenses, EMFL, CNRS UPR 3228, Université Grenoble Alpes, Université Toulouse, Université Toulouse 3, INSA-T, 38042 Grenoble and 31400 Toulouse, France; ‡ Department of Experimental Physics, Faculty of Fundamental Problems of Technology, Wroclaw University of Science and Technology, 50-370 Wroclaw, Poland; § Nanospectroscopy Group and Center for Nanoscience (CeNS), Nano-Institute Munich, Department of Physics, Ludwig-Maximilians-Universität München (LMU), Munich 80539 Germany

**Keywords:** Metal halide perovskites, nanoplatelets, excitons, fine structure splitting, magneto-optical spectroscopy

## Abstract

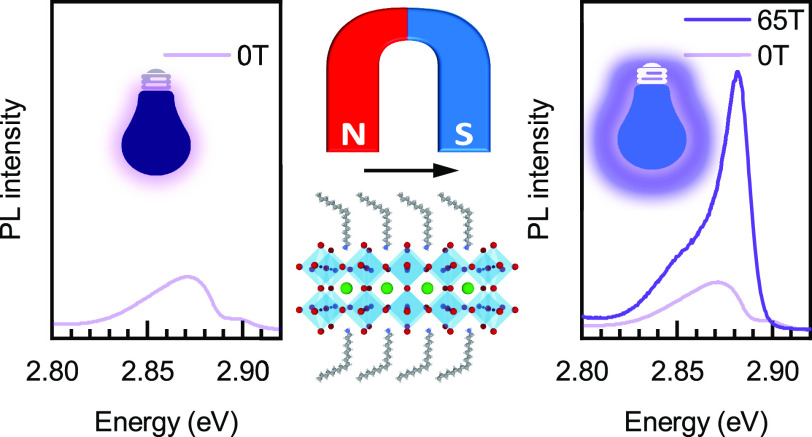

The optimized exploitation of perovskite nanocrystals and nanoplatelets as highly efficient light sources requires a detailed understanding of the energy spacing within the exciton manifold. Dark exciton states are particularly relevant because they represent a channel that reduces radiative efficiency. Here, we apply large in-plane magnetic fields to brighten optically inactive states of CsPbBr_3_-based nanoplatelets for the first time. This approach allows us to access the dark states and directly determine the dark-bright splitting, which reaches 22 meV for the thinnest nanoplatelets. The splitting is significantly less for thicker nanoplatelets due to reduced exciton confinement. Additionally, the form of the magneto-PL spectrum suggests that dark and bright state populations are nonthermalized, which is indicative of a phonon bottleneck in the exciton relaxation process.

The synthesis of colloidal nanocrystals with near-unity photoluminescence (PL) quantum yields has vastly extended the potential of metal halide perovskites for solid-state lighting and display applications.^1−8^ This and their remarkably improved long-term stability^9,10^ have allowed the fabrication of high external quantum efficiency LEDs,^11,12^ low-threshold lasers,^13−15^ and single-photon emitters^16−20^ with long exciton coherence times.^19^ It is possible to template the growth of nanocrystals to form planar, ultrathin perovskite sheets embedded between long organic molecules, which stabilize the colloids, referred to as nanoplatelets.^21−23^ The atomically thin quantum wells shown schematically in Figure 1a, together with the significant difference between the dielectric constants of the organic spacers and inorganic slabs, shift the exciton resonances toward the blue spectral region (essential for the realization of cost-efficient display devices^24^) and vastly increase the exciton binding energy, up to hundreds of meVs.^25−27^

**Figure 1:**
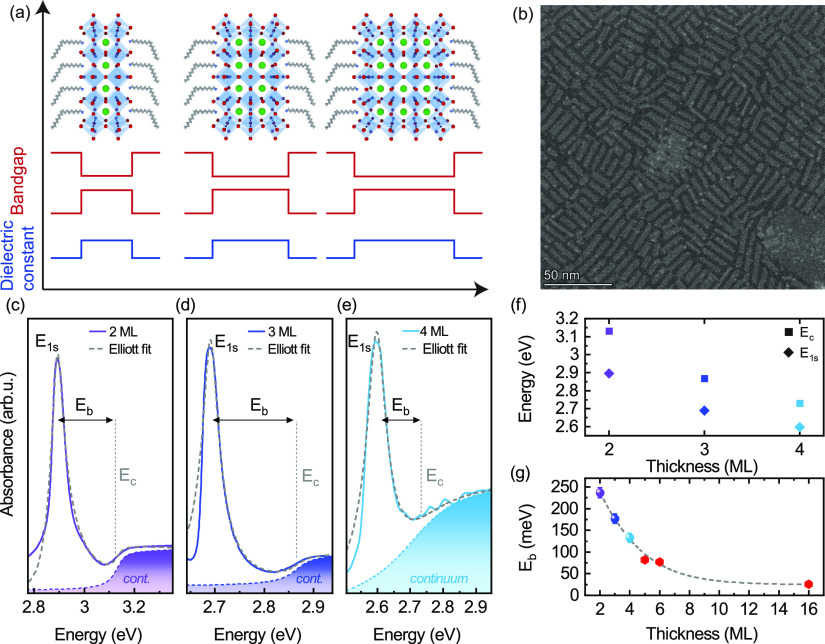
(a) Schematic of the 2, 3, and 4 ML thick nanoplatelets together with the spatial dependence of the band gap and the dielectric constant. (b) STEM images of 3 ML nanoplatelets seen edge-on. (c–e) Absorption spectra of 2–4 ML thick nanoplatelets (continuous lines) fitted by 2D Elliott’s formula (dashed gray lines). The contribution to the absorption spectrum from the continuum is shown as a shaded curve under each spectrum. (f) Energies of the 1s exciton transition (*E*_1s_) and continuum onset (*E*_c_). (g) Measured exciton binding energy (*E*_b_) as a function of nanoplatelet thickness. The red symbols (ML ≥ 5) are values taken from the literature.^49^ The dashed line is drawn as a guide to the eye.

Band-edge exciton states in metal-halide perovskites stem from the Coulomb interaction of a hole residing in the S-like valence band (, ) and an electron residing in the split-off P-like conduction band (, ),^28^ where *J*^e,h^ is the total angular momentum, and *J*_*z*_^e,h^ is its *z*-component. The electron–hole exchange interaction leads to the fine structure splitting^29,30^ with an optically inactive dark state with total angular momentum *J* = 0 and an optically active bright triplet with total angular momentum *J* = 1.^31−34^ An accurate and reliable measurement of the energy spacing within the exciton manifold is not only critical from the fundamental physics viewpoint. The presence of a dark ground state^35^ is potentially detrimental for applications of lead halide perovskites as light emitters. The exciton fine structure of perovskite nanocrystals has been recently the object of extensive investigation.^16,17,19,35−43^ The substantially larger confinement of charge carriers in nanoplatelets greatly enhances the electron–hole exchange interaction^44^ and is expected to increase the splitting between the bright and dark states significantly.^45,46^ The fine structure of perovskite nanoplatelets has received considerably less attention, and experimental efforts have only recently focused on obtaining quantitative measurements.^20,46−48^

By its very nature, a dark state is inherently elusive to spectroscopic measurements. A commonly employed approach to evaluate the splitting between dark and bright excitons relies on the thermal mixing between these two populations and is determined through the temperature dependence of time-resolved PL.^36,46,50−57^ These results are often modeled with rate equations describing the recombination and relaxation processes within a three-level system, where the unknown quantities correspond to relaxation and recombination rates and the energy differences between the levels. However, this method is indirect,^56^ and the three-level model might be oversimplified to describe certain systems accurately. Its results might indeed be affected by the presence of trap states, which introduce significantly smaller activation energies than the real dark bright splitting,^53,54^ the interaction with surface dangling bonds,^55^ or contributions from higher energy states.^46,57^ In fact, instead of treating the dark-bright splitting as a free fitting parameter, in some cases it has been strongly constrained, or even fixed, relying on the outcome of alternative measurement methods.^46,51,56^ In particular, in the case of perovskite nanoplatelets, due to the very large splittings expected within the exciton manifold, temperature-dependent time-resolved measurements alone have been considered unfit to provide a reliable quantitative estimation of the splittings, which were either fixed to values obtained by spectrally- and time-resolved measurements^47^ or to values obtained by theoretical considerations.^46^ This demonstrates the need for an experimental approach that yields straightforwardly the splitting between the dark and bright excitons without the need for background assumptions or complementary experimental methods.

An elegant way to overcome these limitations, providing direct spectroscopic access to dark exciton states, is to apply an in-plane magnetic field, which mixes the bright and dark states.^35,58−61^ In a magnetic field, the dark state is expected to display both a considerable increase of intensity and a red-shift–two features that simultaneously occur only in this excitonic species. Thus, their observation allows us to unambiguously assign the corresponding peak to magnetic field brightened dark excitons without further modeling or assumptions, which are often required in the case of other approaches.^36,37,52,62^

In this Letter, we employ magnetic fields up to 65 T to investigate the exciton fine structure of Cs_*n*–1_Pb_*n*_Br_3*n+*1_ nanoplatelets with a thickness of *n* = 2–4 lead-halide octahedral planes (2–4 ML henceforth), shown schematically in Figure 1a. The magnetic-field-induced brightening enables us to determine the energy separation between dark and bright excitons accurately. Our measurements confirm that the dark state is consistently the lowest-lying exciton state in these nanoplatelets. The measured dark-bright splitting monotonically increases with decreasing thickness of the lead-halide octahedral plane, with values in the range ≃9–22 meV. Additionally, our magneto-optical measurements allow us to shed light on the relaxation processes of excitons in lead halide perovskite nanoplatelets. In these colloidal nanocrystals, discrete, well-spaced states are expected to reduce the phonon scattering efficiency, which has been predicted to lead to a phonon bottleneck effect.^63^ This effect has proven so far to be elusive in most experimental tests.^64−67^ Recently, however, a dependence of the carrier relaxation rate on the nanocrystal size has been reported, which has been considered evidence for the phonon bottleneck in perovskite nanocrystals.^68^ Our magneto-PL spectra, wherein the intensity of the dark and bright excitons suggests that the two populations are not fully thermalized to the lattice temperature, support the phonon bottleneck scenario.

In Figure 1b, we show an annular dark-field scanning transmission electron micrograph (STEM) of an ensemble of 3 ML thick nanoplatelets, demonstrating the high monodispersity of the synthesized sample. The zero-field absorption spectra measured at 5 K of 2, 3, and 4 ML thick nanoplatelets are displayed in Figure 1c–e. The samples were synthesized according to previously published methods.^46,69^ Details on this and the experimental methods are given in the Supporting Information. The spectra display a single excitonic resonance, which we model with a generalized Elliott’s formula to extract the 1s exciton transition energy and the onset of the continuum.^49,70,71^ We plot the obtained 1s and continuum energies in Figure 1f as a function of the nanoplatelet thickness. A thinner inorganic slab increases the confinement of the charge carriers, which enhances the exciton binding energy, as shown in Figure 1g. Here, the exciton binding energy *E*_b_ = *E*_c_ – *E*_1s_ is the difference between the onset of the continuum absorption and the 1s exciton resonance. In weakly confined nanocrystals, with a side length of ≃10–12 nm, the exciton binding energy^49^ is comparable to that of the bulk material.^72^

We now turn to the fine structure of the exciton manifold in perovskite nanoplatelets, schematically presented in the inset of Figure 2. In nanoplatelets, the exchange interaction partially lifts the degeneracy of the bright exciton manifold.^45,46^ Two bright states ϕ_+_, ϕ_–_ are degenerate and have in-plane dipole moments. These states, present in the case of cubic or tetragonal crystal structures,^32,42,45^ couple to circularly polarized light. A lowering of the symmetry of the crystal structures lifts the degeneracy, giving rise to states that couple to two perpendicular linear polarization components. However, if it exists, this splitting is expected to be negligible compared to the dark-bright splitting,^41,42,73^ and we will neglect it in the following discussion. The third bright state, ϕ_*Z*_, has an out-of-plane dipole moment and couples to linearly polarized light, which propagates parallel to the nanoplatelet plane. The optically dark state is generally predicted to be the lowest-lying state.^31,32,35,42,46,58,61^

**Figure 2:**
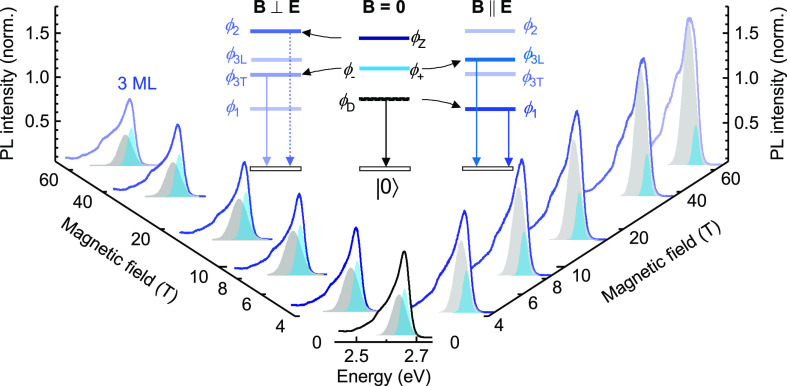
Voigt configuration magneto-PL spectra of 3 ML nanoplatelets up to 65 T at 2 K. Spectra are shown at zero magnetic field (center) and in transversal (left) and longitudinal (right) polarization. Shading highlights the contribution of the PL peaks associated with the bright (blue) and dark excitons (gray). The spectra are normalized to the intensity of the zero magnetic field spectrum. (Inset) Schematic of the exciton manifold showing the selection rules.

In order to access the dark exciton state directly, we apply high in-plane magnetic field in the Voigt geometry. In this configuration, the zero-field exciton states are no longer eigenstates of the system. The new eigenstates can be obtained by considering linear combinations of the zero-field exciton states, with dipole moments oriented longitudinally (labeled L) or transversally (labeled T) to the magnetic field direction
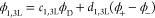
1a
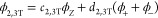
1b

The coefficients are given by
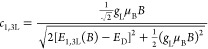
2a
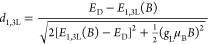
2b
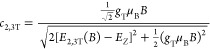
2c
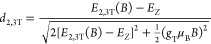
2dwhere *g*_L_ and *g*_T_ are the g-factors for the longitudinal and transverse modes.^58,61,74^ The magnetic field also modifies the energy of the exciton resonances according to
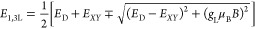
3a
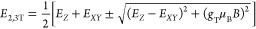
3bwhere *E*_D_, *E*_*XY*_, and *E*_*Z*_ denote the energies of the dark state, the in-plane bright states, and the bright out-of-plane state, respectively.

According to eq 1a the magnetic field transfers the oscillator strength of the bright in-plane states to the dark exciton state. The magnetic field brightened dark state ϕ_1_ couples to longitudinal polarized light (**B** || **E**) and its energy *E*_1_ decreases with increasing magnetic field, as predicted by eq 3a. The magnetic field also mixes the out-of-plane exciton state with the in-plane bright states, see eq 1b. These states couple to transverse polarized light (**B** ⊥ **E**) as indicated in the inset of Figure 2. The ϕ_3T_ state is expected to red shift with increasing magnetic field, as shown by eq 3b.

Before presenting high magnetic field measurements, we focus on the zero-field PL spectrum of nanoplatelets. The PL spectrum of 3 ML thick nanoplatelets exhibits a maximum at ≃2.66 eV and a shoulder on the low energy side (Figure 2, black curve). The spectrum of 2 ML thick nanoplatelets, shown in Figure 3a, consists of the main peak and a weaker high energy peak, which has an energy corresponding to the minimum of the transmission spectrum (∼2.90 eV, see also Supporting Information). This strongly suggests that the high energy peak is related to the recombination of bright excitons (BX), the only species with a strong enough oscillator strength to yield a significant absorption at zero magnetic field. A similar PL line shape, with the simultaneous presence of two peaks attributed to dark and bright exciton states, was previously observed in 2 ML thick nanoplatelets.^47^

**Figure 3:**
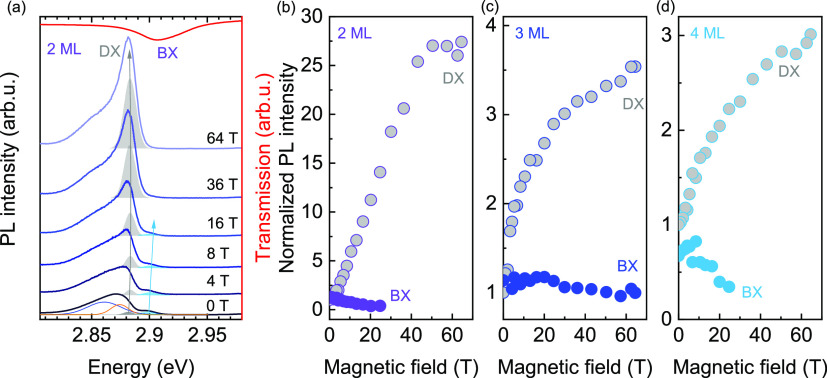
(a) Magneto-PL spectra in the Voigt configuration of 2 ML nanoplatelets, measured in longitudinal polarization (**B** || **E**) up to 64 T at 2 K. The peak attributed to the bright exciton (BX) is shown with blue shading, and the peak attributed to the dark exciton (DX) with gray shading. The vertical arrows are a guide to the eye. The transmission spectrum of the nanoplatelets is plotted in red. (b–d) Magnetic field dependence of the intensity of the dark and bright exciton transitions normalized to the intensity of the dark exciton transition at zero magnetic field for 2–4 ML thick nanoplatelets.

We move now to the description of our magneto-PL measurements, performed at ≃2 K. We acquired magneto-PL spectra in the Voigt geometry with light which is linearly polarized transversally and longitudinally to the applied magnetic field. As seen in Figure 2 for 3 ML thick and Figure 3a for 2 ML thick nanoplatelets, in the longitudinal polarization, the lower energy PL component (gray shade) increases significantly in intensity with increasing magnetic field. We attribute this to the dark exciton (DX), which, as the lowest state, has a large thermal occupation, leading to a significant brightening when the oscillator strength is dramatically increased by the magnetic field-induced mixing of the states. This peak also exhibits a concomitant redshift, as predicted by eq 3a. These concurrent observations represent an unequivocal hallmark of the dipole-forbidden (dark) nature of the transition and provide very strong constraints in analyzing the magneto-PL spectrum. As shown in Figures 2 and 3a, the largest contribution to the PL spectrum in the high field limit stems from the brightened dark exciton peak. Beginning at high magnetic fields, we extract the energy of the dark exciton from the magneto-PL spectra as a function of the magnetic field only to values at which the peak energy can be determined with sufficiently high accuracy. This allows us to reliably fit the magnetic field dependence of the dark exciton energy using eq 3a. By extrapolating this dependence to zero field, we can readily obtain the energy of the dark exciton at zero magnetic field without the need for analyzing in detail the corresponding zero-field PL spectrum. More details on the analysis of the magneto-PL spectra are provided in the Supporting Information.

Extrapolating the position of dark exciton peak to the low magnetic field limit, we note that it is also weakly present in the zero-field PL spectrum (Figure 2 and Figure 3a). This is consistent with prior reports on nanoplatelets^46,47^ and Ruddlesden–Popper perovskites^31,75−77^ and is probably related to a mixing of the dark and bright states due to spin–orbit interaction,^31,76,78,79^ crystal distortions^61,75,77^ or phonon-assisted transitions.^61,75^ The higher energy PL band blue shifts and loses intensity with increasing in-plane magnetic field, as predicted by eq 3a. This confirms our assignment of this feature to emission from bright in-plane exciton states. More details on the fitting of the magneto-PL spectra, together with qualitatively similar magneto-PL of the 4 ML thick nanoplatelets, are provided in the Supporting Information.

It is important to note that our results suggest that the dark exciton is only partially responsible for the low energy PL spectrum at zero magnetic field. For example, for the 2 ML thick nanoplatelets, to fully describe the PL, we need to add additional transitions with maxima at ∼2.86 and ∼2.875 eV (dark blue and orange curves, see Figure 3a). The energy and emission intensity of these peaks are weakly dependent on the magnetic field strength. This suggests that these are related to the random potential landscape, due to the presence of unsaturated bonds at the boundary between the inorganic slabs and the ligands or at lattice defects, which can localize dark^79^ or bright^77^ excitons. These results suggest that the PL spectrum of CsPbBr_3_-based nanoplatelets is richer than initially thought, with features attributed to multiple bright, dark, and localized excitonic transitions. Similar considerations apply also to thicker nanoplatelets, as shown in the Supporting Information, where in Experimental Methods we also explain the origin of the differences between some of the spectral features reported here and those of pristine samples.

In the transversal polarization, we observe an overall reduction in the intensity of the high energy PL peak, accompanied by a slight red-shift, as shown in Figure 2 and in the analysis presented in the Supporting Information. This is expected for ϕ_3T_ since the mixing of the bright in-plane states with the out-of-plane state (see eq 1b) reduces the oscillator strength of the in-plane states. Additionally, the red-shift of this peak with increasing magnetic field is consistent with eq 3b (see Supporting Information for the complete magnetic field dependence of the PL energy on the magnetic field in transversal polarization). The large spectral broadening, together with the low thermal occupation, prevents us from observing the magnetic field brightened out-of-plane state (ϕ_2_) at higher energies. The significantly lower thermal occupation of the out-of-plane state cannot be compensated by the transfer of oscillator strength from the in-plane states. This conclusion is also supported by theoretical considerations on the order of states in the bright exciton manifold.^46^ The relaxed selection rules for the dark state allow for its observation also in the transversal polarization,^75^ as shown in Figure 2 and discussed in the Supporting Information.

To corroborate our PL peak assignment, we analyze the intensities of the bright and dark exciton states (Figure 3b–d). We normalize all the peaks to the intensity of the dark exciton peak at zero magnetic field. The intensity of the dark state emission increases considerably, while that of the bright states decreases slightly with increasing magnetic field. This is fully consistent with the expected transfer of oscillator strength due to the magnetic field induced bright-dark mixing in the Voigt geometry. The 2 ML thick nanoplatelets exhibit the largest increase in PL intensity. This might appear surprising at first sight, considering that eq 2b predicts that a large dark-bright energy difference corresponds to a small gain in the dark excitons oscillator strength, as shown in the Supporting Information. However, the PL intensity is related to the product of the state occupation and the oscillator strength. Accordingly, a significantly larger population of the dark exciton state of the thinnest nanoplatelets can account for its larger magneto-PL intensity, as we discuss below in detail.

The magnetic field dependence of the energy of the bright and dark states, obtained by fitting the magneto-transmission spectra (see Supporting Information), is shown in Figure 4a–c. The predicted evolution using eq 3a reproduces well the observed blue and red-shifts and allows us to extract the dark-bright splitting and a rough estimate of the longitudinal state’s g-factor *g*_L_ ≃ 1.2–2.3 (see Supporting Information for details). The obtained dark-bright splitting, shown in Figure 4d, increases with decreasing thickness of the inorganic slab of nanoplatelets from ∼9 meV for the 4 ML nanoplatelets to ∼21 meV for the 2 ML nanoplatelets. The increase, as noted previously, is due to the larger dielectric and quantum confinement in the colloidal perovskite quantum wells.^30,34,45^ The splittings reported here are slightly smaller than those measured in nanoplatelets synthesized with a very similar approach,^46^ possibly due to a slightly larger in-plane size of the nanoplatelets investigated in our study and a concomitantly reduced in-plane confinement. However, the values are still considerably larger than the dark-bright splitting of conventional nanocubes,^35,36^ which exhibit far less confinement.^44^

**Figure 4:**
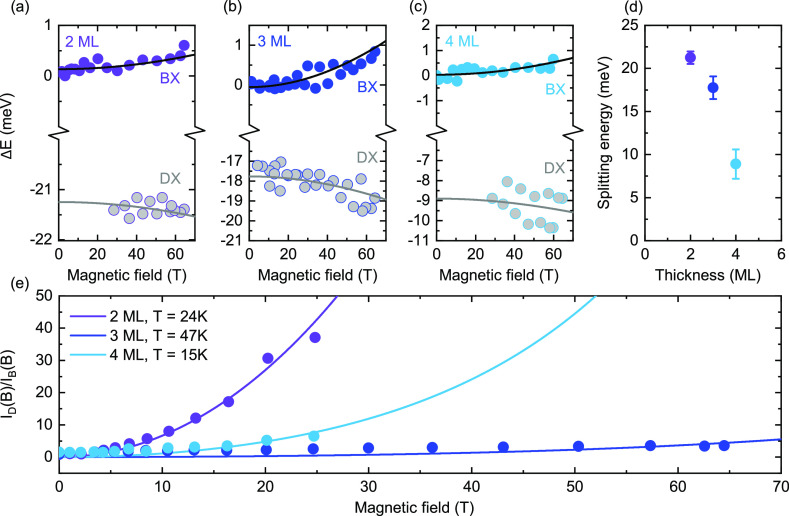
(a–c) Magnetic field dependence of the energies of the dark and bright exciton transitions relative to the zero-field transition energy of the bright state Δ*E* for 2–4 ML nanoplatelets. The lines are fits of the data to eq 3a. (d) Measured bright-dark splitting as a function of nanoplatelet thickness. (e) PL intensity ratio between dark and bright exciton states for the three nanoplatelet thicknesses investigated as a function of the applied magnetic field. Full circles represent experimental points. The curves are calculated using eq 4, and the assumed temperature is indicated in the inset.

In Figure 4e, we plot the magnetic field dependence of the ratio between the intensity of the dark states, *I*_D_ and the bright states, *I*_B_, which we calculate as
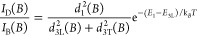
4where the Boltzmann factor accounts for the population ratio between the dark and bright states.^61^ For simplicity, we neglect here the presence of localized exciton states. Due to the considerably more populated dark state in the 2 ML thick nanoplatelets, the PL intensity of the dark state is stronger in thinner nanoplatelets, as shown in Figure 4e. Importantly, to reproduce the experimental data, we had to use an effective exciton temperature of *T* = 15–47 K, as indicated in the inset of Figure 4e. These temperatures are at least an order of magnitude higher than the lattice temperature of ∼2 K. This mismatch between the lattice temperature and the effective exciton temperature suggests that the exciton population is not fully thermalized at the moment of the recombination.^61^

This observation can be explained in light of the energy level structure of the exciton manifold, in combination with the peculiar exciton–phonon coupling in metal halide perovskites. Due to the greatly enhanced exchange interaction in a strongly confined system,^44^ the splittings in the exciton manifold exceed the energies of longitudinal optical (LO) phonons, with energies in the ∼15–33 meV range,^69,80,81^ to which excitons can couple to efficiently dissipate excess energy. In the case of the 4 ML thick nanoplatelets, a more efficient exciton relaxation to the dark state might be driven by an 8 meV LO phonon,^80^ to which, however, carriers do not couple very efficiently.^69^ This energy mismatch between LO phonons and dark-bright splittings, together with the virtually negligible coupling of carriers to acoustic phonons in metal halide perovskites,^82^ leads to the highly nonthermalized exciton population visible in Figure 4e and supports the presence of a phonon bottleneck effect in strongly confined perovskite nanocrystals.^68^

In conclusion, we have used large in-plane magnetic fields to accurately extract the splitting between the dark and the bright exciton states of CsPbBr_3_-based nanoplatelets. In this configuration, the optically inactive states are brightened by the applied magnetic field and become the strongest contribution to the PL spectra while simultaneously red shifting with increasing magnetic field. The combination of these two observations allows us not only to accurately determine the splitting between the dark and bright excitons, even when this is considerably smaller than the inhomogeneous broadening of the PL spectrum, but also to reveal the presence of additional PL bands, possibly related to the localization of excitons. This approach, applied here for the first time to perovskite-based nanoplatelets, enabled us to confirm that the lowest-lying exciton state is optically dark and to determine the energy spacing between optically dark and optically bright states. The measured decrease in the dark-bright splitting with increasing thickness of the nanoplatelets nicely reflects the expected increase in carrier confinement as the number of lead-halide octahedral planes is decreased. The energy splittings and magneto-PL intensities determined experimentally suggest that the dark and bright excitons are not fully thermalized with the crystal lattice. We attribute this observation to the mismatch between the large splittings within the exciton manifold and the energies of the LO phonons in metal halide perovskites. This, in combination with the poor coupling with acoustic phonons, leads to the observed nonthermalized exciton distribution, suggestive of the phonon bottleneck effect.

## Supporting Information Available

Experimental methods: nanoplatelets’ synthesis and magneto-optical spectroscopy; note on the nanoplatelets’ orientation; zero-field transmission and PL spectra; additional details concerning the analysis of the magneto-optical spectra in the longitudinal and transversal polarization (PDF)
