# Supplementary material for: Thickness-Dependent Dark-Bright Exciton Splitting and Phonon Bottleneck in CsPbBr3-Based Nanoplatelets Revealed via Magneto-Optical Spectroscopy
Source: Nano Lett. 2022 Aug 29;22(17):7011–9. doi: 10.1021/acs.nanolett.2c01826 (PMC9479212; doi:10.1021/acs.nanolett.2c01826)
Supplement: Supplementary file 1 — nl2c01826_si_001.pdf [file nl2c01826_si_001.pdf]

# Supplementary Information
# Thickness-dependent dark-bright exciton splitting and phonon bottleneck in $CsPbBr_3$-based nanoplatelets revealed via magneto-optical spectroscopy

Shuli Wang,[1] Mateusz Dyksik,[2] Carola Lampe,[3] Moritz Gramlich,[3] Duncan K. Maude,[1] Michał Baranowski,[2] Alexander S. Urban,[3,*] Paulina Plochocka,[1,2,†] and Alessandro Surrente[2,‡]

[1]*Laboratoire National des Champs Magnétiques Intenses, EMFL, CNRS UPR 3228, Université Grenoble Alpes, Université Toulouse, Université Toulouse 3, INSA-T, 38042 Grenoble and 31400 Toulouse, France*

[2]*Department of Experimental Physics, Faculty of Fundamental Problems of Technology, Wroclaw University of Science and Technology, 50-370 Wroclaw, Poland*

[3]*Nanospectroscopy Group, Nano-Institute Munich, Department of Physics, Ludwig-Maximilians-Universität München (LMU), Munich, 80539 Germany*

(Dated: August 27, 2022)

## I. EXPERIMENTAL METHODS

### A. Nanoplatelets synthesis

The colloidal $CsPbBr_3$ nanoplatelets are synthesized by a reprecipitation method at room temperature, and the thickness from 2 lead-halide octahedra planes (ML) to 4ML can be precisely controlled by altering the molar ratio of the precursors. First of all, 0.1 mmol $Cs_2CO_3$ (cesium carbonate, 99 %) powder was dissolved in 10 mL oleic acid at 100 °C under continuous stirring to obtain the Cs-oleate precursor. Similarly, Pb-$Br_2$ precusors was prepared by dissolving 0.1 mmol Pb-$Br_2$ (lead(II) bromide, $\geq$ 98 %) powder in 10 mL toluene at 100 °C, as well as 100 µL each of oleylamine and oleic acid was added to solubilize Pb-$Br_2$ better in toluene. Then in order to obtain 2 ML nanoplatelets, 150 µL Cs-oleate precursor solution was dropped into 3 mL $PbBr_2$ precursor solution under vigorous stirring at room temperature. After 5 s, acetone was added into the mixed solution to initiate the formation of 2ML nanoplatelets. After 1 min of stirring, the solution was centrifuged at 4000 rpm for 3 min then the precipitate redispersed in 2 mL of hexane. It is worth noting that the thickness of the nanoplatelets can be precisely determined by the ratio of the precursors. For 3ML, the volume of the Pb-$Br_2$ precusors solution is 1.5 mL and for 4ML is 1.2 mL, synchronously keeping other steps and conditions as the same as the synthesis of 2ML thick nanoplatelets.

### B. Magneto-optical spectroscopy

The magneto-optical measurements were performed in a high magnetic field facility, where the preparation of fresh samples is impossible due to the lack of specific equipment. This implied that the samples had to be prepared and shipped to the high field facility. Even though all the possible precautions were taken to minimize the time between the synthesis and the optical measurements, the time elapsed before the dispersions were drop-cast might have induced the formation of defects or some additional growth of the nanocrystal due to residual precursors inadvertently left in the dispersion. This could explain the discrepancies between the spectra measured in the high magnetic field facility and those of fresh samples

* urban@lmu.de
† paulina.plochocka@lncmi.cnrs.fr
‡ alessandro.surrente@pwr.edu.pl

measured immediately after the synthesis [1]. After shipment to the magnetic field facility, the nanoplatelets were dropcast on glass substrates, which were ultrasonically cleaned in ethanol. The substrates were dried with dust free air. The dropcast substrates were installed with the plane of the substrate parallel to the magnetic field vector in the sample space of a probe, which fitted into the variable temperature insert of a helium cryostat mounted at the center of a liquid nitrogen cooled resistive pulsed magnet. The pulsed magnet was powered by a 14 MJ capacitor bank, which can generate pulsed magnetic field up to 65 T, with a pulse duration $\sim 500$ ms. The photoluminescence (PL) was excited with a continuous wave laser emitting at 405 nm. The laser was coupled in a multimode optical fiber, which guided the light to the sample. The PL was coupled in an output optical fiber, which was used to direct the signal to the detection path. The PL was spectrally dispersed and detected by a monochromator equipped with diffraction gratings and a nitrogen-cooled CCD camera. The measurements were performed in the Voigt configuration, with the **c** axis of the sample perpendicular to the magnetic field and parallel to the **k** vector of the light. The linear polarization was resolved in situ by a broadband polarizer. Transmission spectra were obtained in the same geometry. For transmission measurements, a xenon lamp was used as the broad band light source for 2 ML and 3 ML thick nanoplatelets, while a tungsten halogen lamp was used for 4 ML thick nanoplatelets. All magneto-optical measurements were performed at 2 K.

## II. NOTE ON THE NANOPLATELET ORIENTATION

During the drop-casting process, no specific effort was undertaken to force the orientation of the nanoplatelets in a specific direction. It is thus likely that a fraction of the nanoplatelets in the ensemble lie parallel to the substrate. These nanocrystals are indeed oriented with the magnetic field perpendicular to the c axis, thus in the Voigt geometry. The nanoplatelets oriented edge-on, as in Fig. 1(b) of the main text, will have a random direction with respect to the applied magnetic field. For these nanoplatelets, the magnetic field $B$ can be decomposed in an in-plane component $B_{\mathrm{V}} = B \sin\theta$ and in an out-of-plane component $B_{\mathrm{F}} = B \cos\theta$, where $\theta$ is the polar angle between the **c** axis of the nanoplatelet and the magnetic field vector. These magnetic field components will act as if the nanoplatelet was purely in a Voigt (Faraday) configuration, with an effective magnetic field of $B_{\mathrm{V}}$ ($B_{\mathrm{F}}$) [2].

In our experimental setup, we use a linear polarizer mounted parallel and perpendicular to the magnetic field vector to facilitate the identification of spectral features. In the former configuration, for the nanoplatelets which lie flat on the substrate, the transmitted light corresponds to the longitudinal exciton state, defined in the main text. For the edge-on nanoplatelets, the signal transmitted by the polarizer will be mainly contributed by nanoplatelets with the **c** axis close to parallel to the magnetic field vector (close to Faraday configuration, $\theta \approx 0^{\circ}$, with the dipole moment of the out-of-plane exciton parallel to the direction of the polarizer). In this case, the dark exciton $\phi_{\mathrm{D}}$ will couple with the out-of plane state $\phi_Z$. The energy of the magnetic-field brightened dark exciton is $E = \frac{1}{2}\left[E_Z + E_{\mathrm{D}} - \sqrt{(E_Z - E_{\mathrm{D}})^2 + (g_{\mathrm{D}}\mu_{\mathrm{B}}B)^2}\right]$, where $E_Z$ and $E_{\mathrm{D}}$ are the zero-field energies of the out-of-plane exciton and dark exciton, $g_{\mathrm{D}}$ is the g-factor of the dark exciton in the Faraday configuration, and $\mu_{\mathrm{B}}$ is the Bohr magneton [3]. This expression is very similar to Eq. (3a) of the main text, and the shift rate of the dark exciton is expected to be very similar to that of the dark exciton in the Voigt configuration. The nanoplatelets at intermediate angles will be subjected to an effectively smaller in-plane magnetic field, and exhibit a smaller energy shift of the dark exciton, which might result in a slight asymmetry of the peak related to the dark exciton on the high energy side. They can be, however, optically active in the transversal polarization, as briefly discussed in Sec. IV B.

## III. ZERO FIELD TRANSMISSION AND PL SPECTRA

In Fig. S1(a,b,c), we show transmission and PL spectra of nanoplatelets having thickness of 2, 3 and 4 lead-halide octahedra planes (2ML, 3ML and 4ML henceforth), respectively, measured at zero magnetic field. The high energy peak of the PL spectrum of 2ML nanoplatelets, attributed to the recombination of the bright exciton manifold, is very close to the minimum of the transmission spectrum ($\sim 6$ meV shift), as seen in Fig. S1(a). The shift between the PL and transmission spectrum is considerably larger for 3 and 4ML thick nanoplatelets ($\sim 46$ meV and $\sim 40$ meV shift), as shown in Fig. S1(b,c), respectively.

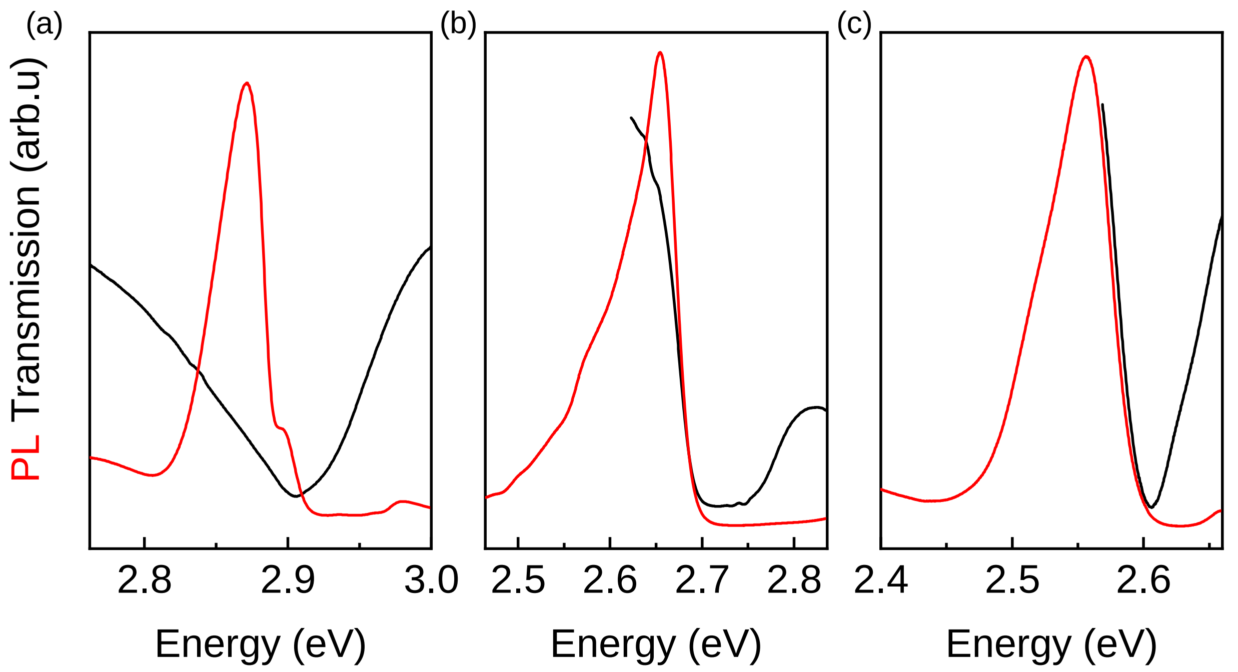


FIG. S1. Low temperature PL (red) and transmission (black) spectra of (a) 2 ML, (b) 3ML and (c) 4ML thick nanoplatelets.

## IV. ANALYSIS OF MAGNETO-OPTICAL SPECTRA

### A. Magneto-optical spectra in the longitudinal polarization

In Fig. S2, we show the global evolution of the PL spectrum as a function of the applied in-plane magnetic field. The peak attributed to the dark state increases its intensity considerably and red shifts with increasing magnetic field, while the intensity of the peak attributed to bright excitons decreases.

In Fig. S3, we show selected PL spectra of nanoplatelets of different thicknesses, resolved in the longitudinal linear polarization ($\mathbf{E} \parallel \mathbf{B}$), at three selected values of magnetic field together with the different peaks used to fit the data and extract the energies shown in the main text. We begin the analysis of the magnetoPL spectrum by the PL spectra measured at high magnetic field, where the contribution of the dark exciton is very pronounced. We keep track of this peak with decreasing magnetic field only up to values at which the energy of the peak can be determined with sufficiently high accuracy, which are the values shown in Fig. 4 of the main text. The magnetic field dependence of the energy of the dark states reported in Fig. 4 of the main text is then extrapolated to zero field by means of Eq. (3a)

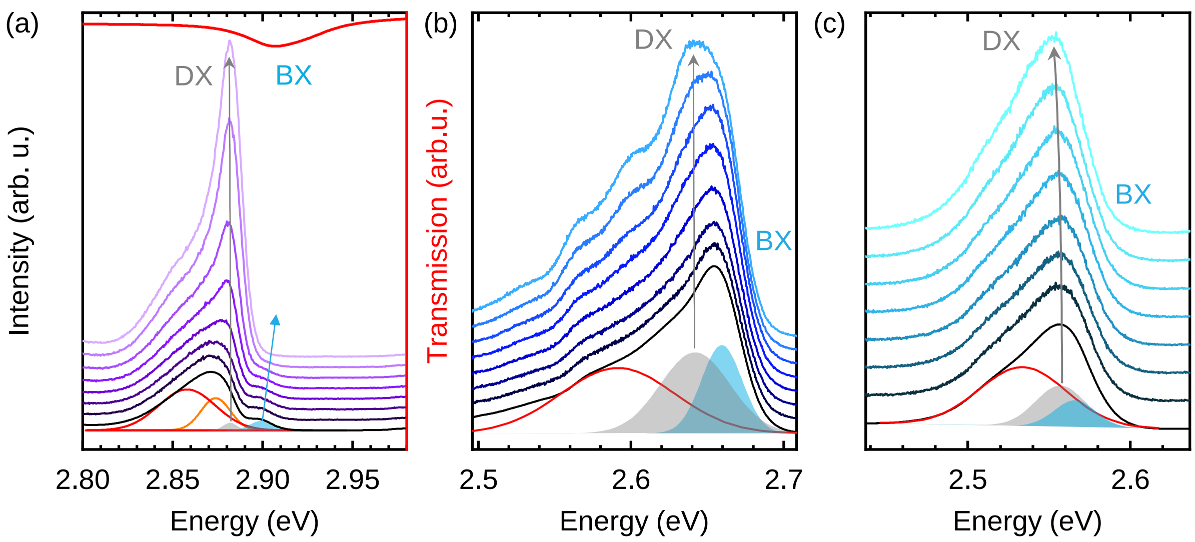


FIG. S2. PL spectra ($\mathbf{E} \parallel \mathbf{B}$) at the indicated magnetic fields of (a) 2, (b) 3, and (c) 4ML thick nanoplatelets. Multiple Gaussian fits are also shown for the zero field spectrum. In panel (a), the corresponding zero-field transmission spectrum is also shown.

of the main text. The zero field PL spectra, which we show also in Fig. S3, is then fitted by fixing the energy of the dark state. To account for the line shape of the zero-field PL spectrum of the different samples, we had to use a peak on the high energy side of the dark exciton, which we attribute to the bright exciton PL, shown in blue in Fig. S3. The additional Gaussian curves used on the low energy side of the dark exciton in the fitting might be related to the presence of trap states, possibly related to unsaturated bonds, or to the presence of residual concentration of thicker nanoplatelets, as discussed in the main text.

At zero magnetic field, 2ML thick nanoplatelets exhibit a high energy peak, as depicted in Fig. S3(a), which we attribute to the contribution of the bright exciton manifold to the PL spectrum. The dark exciton state is shown as a dark, shaded peak. With increasing magnetic field, the intensity of the high energy peak decreases rapidly, while the originally dark state becomes the most intense peak. The magnetoPL of 3ML thick nanoplatelets is shown in Fig. S3(b). In this case, the zero field spectrum consists of the contribution of bright and dark excitons. At high magnetic field, the dark exciton becomes the predominant emission peak. A similar trend can be observed in the magnetoPL spectrum of 4ML thick nanoplatelets, shown in Fig. S3(c). At zero magnetic field, the spectrum can be described with a very weak,

high energy band attributed to the recombination of bright excitons. Already at zero field, the dark exciton contribution is larger, with a low energy peak possibly related to residuals of thicker nanoplatelets in the analyzed sample. Upon the increase of magnetic field, the intensity of the bright exciton band decreases and its contribution to the PL spectrum at 30 T or higher is negligible.

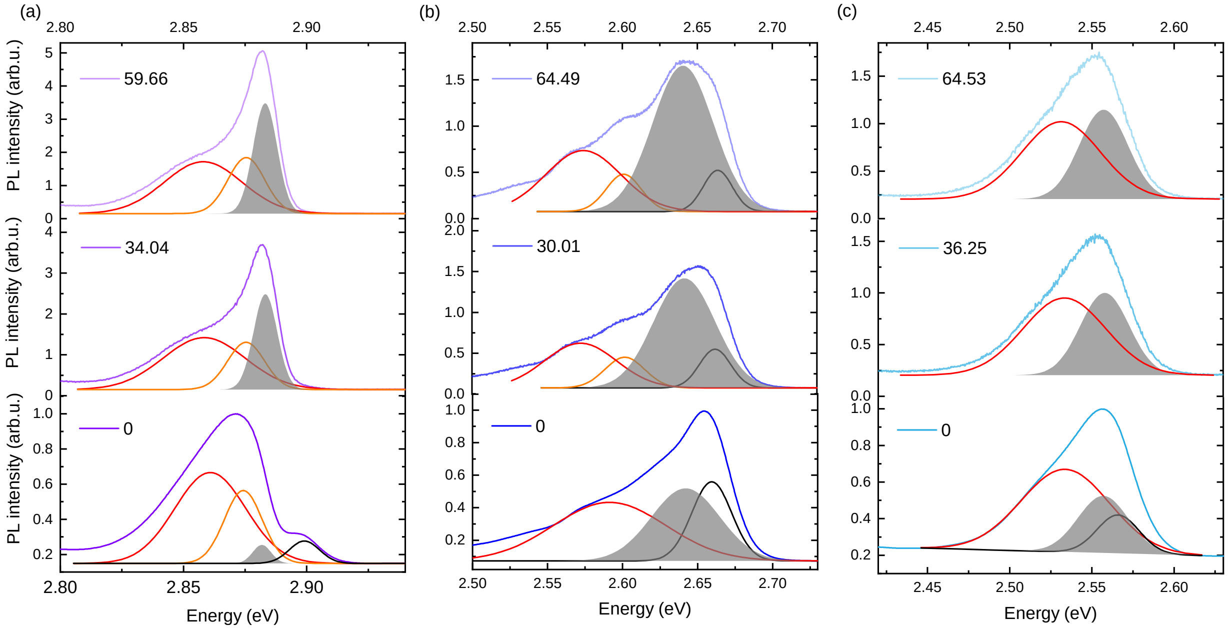


FIG. S3. PL spectra ($\mathbf{E} \parallel \mathbf{B}$) at 0 T, $\sim$ 30 T and $\sim$ 60 T of (a) 2, (b) 3, and (c) 4ML thick nanoplatelets. Multiple Gaussian fits are also shown. The bright state is shown with a blue curve, the dark state at zero field is represented by a dark, shaded curve. Additional peaks are shown in red and grey.

We show magneto-transmission spectra resolved in the longitudinal polarization ($\mathbf{E} \parallel \mathbf{B}$) of 2ML, 3ML and 4ML thick nanoplatelets measured at 0 T, $\sim$ 30 T, $\gtrsim$ 60 T in Fig. S4(a,b,c), respectively. Due to the small shifts, to reliably extract the magnetic field dependence of the absorption energy, we calculated the ratioed spectrum $T(B)/T(0)$, which shows resonance features we use to determine the magnetic field induced shifts without recurring to a standard fitting procedure [4]. The ratioed spectra of 2 and 3ML thick nanoplatelets are shown in the insets of Fig. S4(a,b), respectively. In the case of 4ML thick nanoplatelets, the magnetic field induced shift can be accurately caught by a Gaussian fitting of the transmission spectrum, as shown in Fig. S4(c). The line shape of the magnetotransmission spectra does not change as a function of the magnetic field, which suggests that the transmission feature is mainly

related to the absorption of bright exciton states. These energies, renormalized as explained in the main text, represent the upper branches of Fig. 4 of the main text.

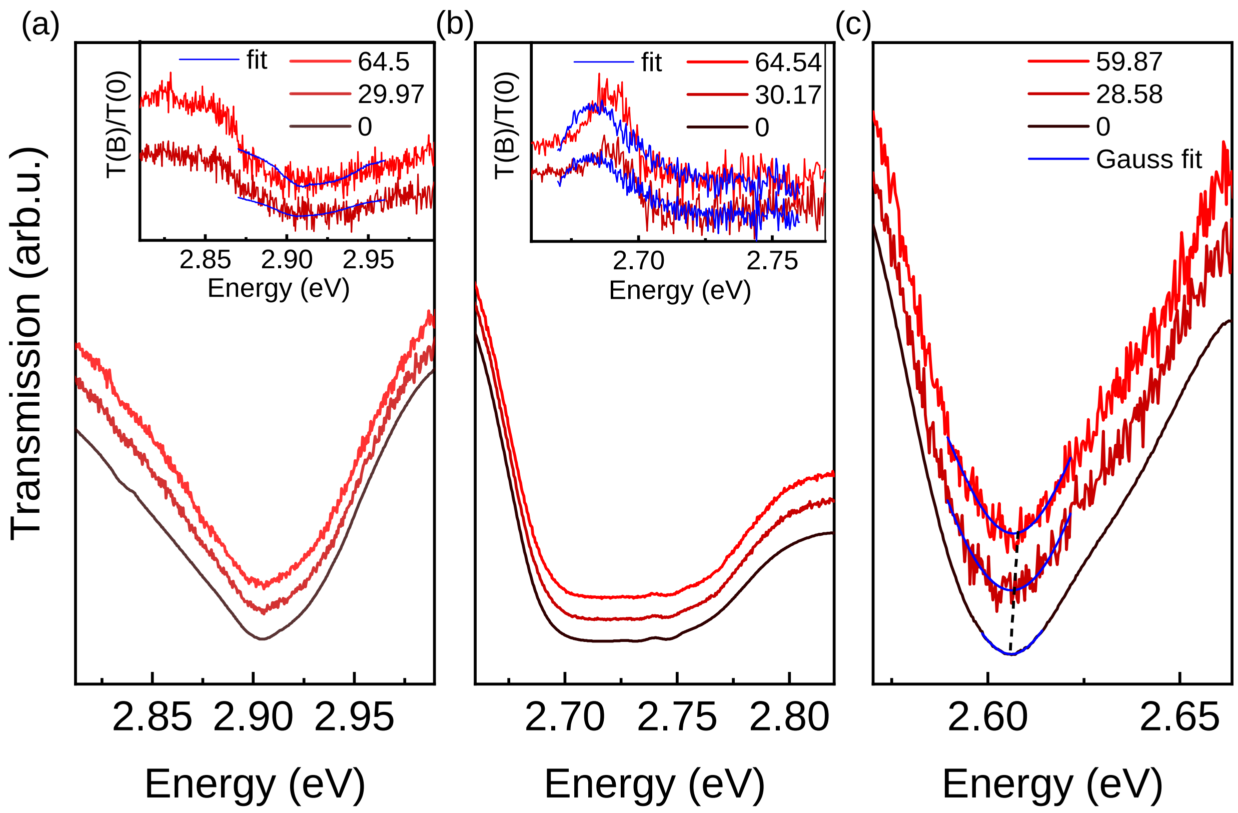


FIG. S4. Transmission spectra of (a) 2ML, (b) 3ML, (c) 4ML thick nanoplatelets measured at the indicated values of magnetic field for **E** ∥ **B**. In panel (a), (b), the inset shows the ratioed spectrum obtained by dividing the high field by the zero field transmission spectrum, which allowed us to determine the magnetic field-induced shift of the exciton resonance.

The fitting of the data presented in Fig. 4 of the main text with Eq. (3a) allows us not only to determine accurately the splitting between the dark and the bright states, but also the Landé g-factor of the longitudinal states $g_{\mathrm{L}}$ in the Voigt configuration. The values of this quantity for all thicknesses are reported in table I. The g-factor of longitudinal transitions of bulk $CsPbBr_3$ has been difficult to extract, due to the relatively large dark-bright splitting in this material [5]. Prior magnetic field measurements allowed to determine experimentally the g-factor of the transversal transition in the Voigt configuration, measured to be $\sim 2.3$ [5]. More recently, g-factors of electrons and holes in the Voigt geometry have been measured in bulk $CsPbBr_3$ [6]. The g-factor of the exciton for the longitudinal transition can be

estimated as $g_L = g_{e\perp} - g_{h\perp}$, where $g_{e\perp}$ and $g_{h\perp}$ are the g-factors of the electron and of the hole in the Voigt configuration, respectively. Based on the g-factor of resident carriers recently reported [6], one can calculate the effective g-factor of the dark exciton to be $\sim 0.85$. The values reported in table I do not differ significantly from the corresponding value of the bulk material, as observed for two-dimensional perovskite compounds [7]. The dependence of the g-factor on the electronic band gap [6, 8] alone does not allow to explain the dependence of the g-factor on the thickness of the inorganic slab, which suggests that the carrier confinement might play a role [9].

TABLE I. Summary of measured bright-dark splittings ($\Delta$) and effective $g$-factors for longitudinal ($g_L = g_{e\perp} - g_{h\perp}$) in Voigt geometry.

| Thickness | $\Delta$ (meV) | $g_L$ |
|---|---|---|
| 2ML | $21.25 \pm 0.44$ | $1.23 \pm 0.17$ |
| 3ML | $17.76 \pm 1.31$ | $2.31 \pm 0.14$ |
| 4ML | $8.90 \pm 1.70$ | $1.27 \pm 0.32$ |

Fig. 3 of the main text shows that the intensity of the dark state increases with increasing magnetic field more prominently for 2ML thick nanoplatelets and progressively less for thicker nanoplatelets. Based on the predictions of Eq. (3b), which describes the oscillator strength of the longitudinal exciton mode as a function of the applied in-plane magnetic field, this trend is counterintuitive. Equation (3b) suggests that the smaller the energy difference between the dark and the bright excitons, the larger increase of the oscillator strength of the dark exciton. Based on the experimentally determined energy splittings and effective g-factors, we plot the ratio between the oscillator strength of the dark state $d_1^2$ and the bright states $d_{3L}^2 + d_{3T}^2$ as a function of the applied magnetic field, which we show in Fig. S5. As expected, the largest increase of the ratio is exhibited by the 4ML thick nanoplatelets, for which the energy separation between the dark and bright exciton states is smallest. This however does not translate directly in a corresponding increase of the PL intensities of the darks states for different thicknesses of the lead-halide slab, due to the different exciton populations and relaxation rates, as we highlight in the main text.

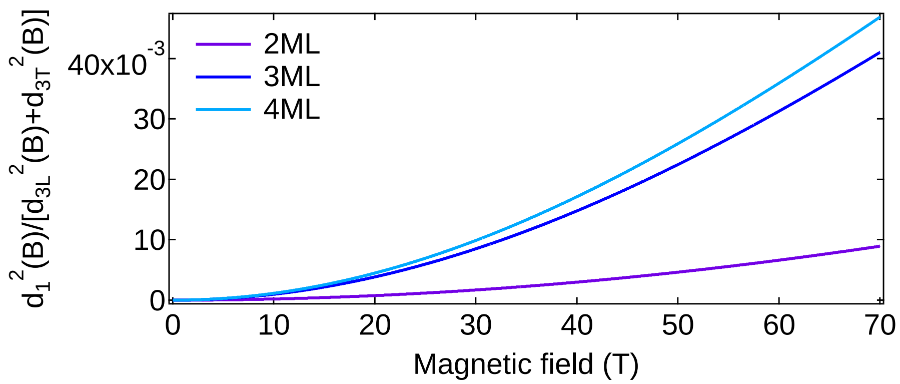


FIG. S5. Ratio of the oscillator strength of the magnetic field brightened dark state $d_1^2$ to that of the bright states $d_{3\mathrm{L}}^2 + d_{3\mathrm{T}}^2$ as a function of the applied magnetic field.

### B. Magneto-optical spectra in the transversal polarization

We performed magnetoPL measurements of nanoplatelets in the transversal polarization ($\mathbf{E} \perp \mathbf{B}$). As shown in the main text, in the presence of an in-plane magnetic field, light polarized perpendicular to the direction of the applied magnetic field can couple to exciton states obtained as linear combinations of the in-plane and out-of-plane bright excitons [3]. In Fig. S6(a,b,c), we show the magnetoPL spectra of 2, 3 and 4ML thick nanoplatelets at $\sim 30\,\mathrm{T}$ and $64\,\mathrm{T}$. In the case of 2ML thick nanoplatelets, the line shape reported in Fig. S6(a) is surprisingly similar to that observed in the longitudinal polarization ($\mathbf{E} \parallel \mathbf{B}$). This might be related to the presence of a fraction of nanoplatelets oriented perpendicular to the substrate during dropcasting [10]. For these nanoplatelets, there is a magnetic field component parallel to the **c** axis and, in this configuration, the dark state couples with the out-of-plane bright state, thus gaining oscillator strength [3]. The presence of a tilt angle between the **c** axis and the magnetic field (and thus the polarizer) allows some PL in the detection path. This explains the appearance of a peak at high magnetic field which has an energy and line shape similar to the peak attributed to the dark exciton in the main text, but with half the intensity measured in the longitudinal polarization. In the case of 3 and 4ML thick nanoplatelets, shown in Fig. S6(b,c), respectively, the originally bright in-plane exciton couples to the out-of-plane exciton in the transversal polarization, as described by Eq. (1b) of the main text.

In Fig. S7(a), we show the dependence of the bright energy state on the magnetic field

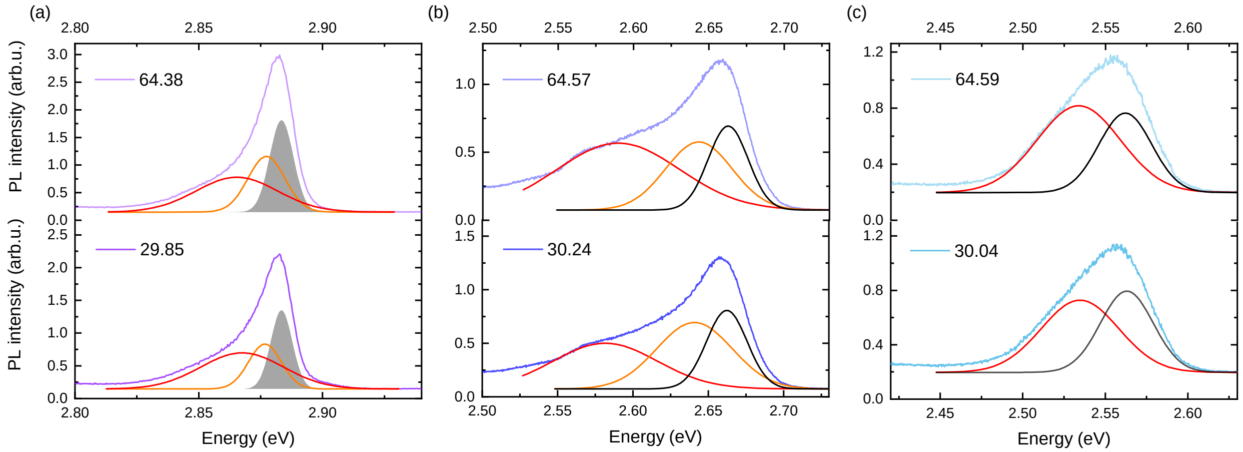


FIG. S6. PL spectra ($\mathbf{E} \perp \mathbf{B}$) at $\sim 30\,\mathrm{T}$ and $\sim 64\,\mathrm{T}$ of (a) 2, (b) 3, and (c) 4ML thick nanoplatelets. Multiple Gaussian fits are also shown. The bright state is shown with a blue curve. Additional peaks are shown in red and grey.

for 3 and 4ML thick nanoplatelets for PL spectra measured in the transversal polarization ($\mathbf{E} \perp \mathbf{B}$). We observe a global red shift of the signal with increasing magnetic field. This reflects the negative branch of the transversal exciton states [see Eq. (3b) of the main text], where excitons relax before recombining radiatively. We also observe a decreased intensity with increasing magnetic field for 3 and 4ML thick nanoplatelets, as shown in Fig. S7(b,c), respectively. This observation can be explained with the mixing of the in-plane bright exciton states with the out-of-plane bright exciton state, in the hypothesis that the latter resides at higher energy [3].

In S8(a), we plot the magnetic field dependence of the peak attributed to the dark exciton of the 3ML thick nanoplatelets as a function of the magnetic field in the transversal polarization ($\mathbf{E} \perp \mathbf{B}$). According to the selection rules described in the main text, the dark state should not couple to the transversal polarization. The PL of nominally dipole forbidden excitons have been investigated in the Voigt configuration, where transitions forbidden by selection rules have been observed [11], due to relaxed selection rules in the presence of spin-orbit interaction [12–15], crystal distortions [7, 11, 16] or phonon-assisted transitions [7, 11]. In the transversal polarization, the peak at $\sim 2.6\,\mathrm{eV}$ displays a blue shift with increasing magnetic field. This behaviour resembles the expected behaviour of the out-of-plane bright state in the transversal polarization. This might suggest that the mixing with this state might be one of the mechanisms responsible for the observation of the PL of the dark state

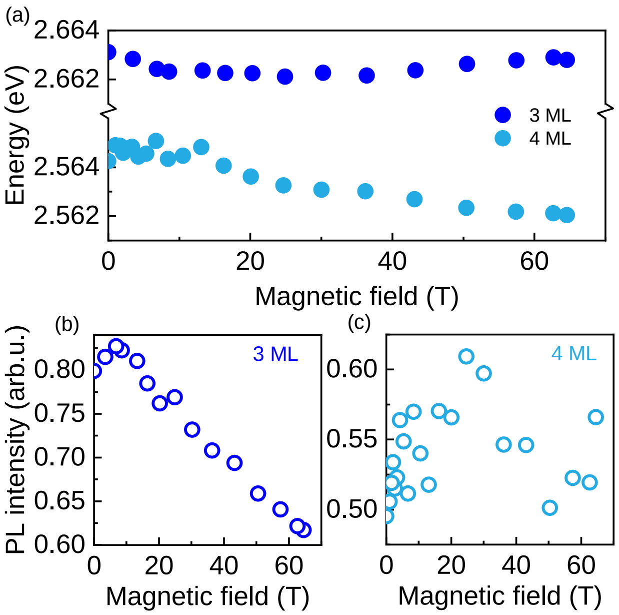


FIG. S7. (a) Energy of bright exciton state as a function of the magnetic field of 3 and 4ML thick nanoplatelets in the transversal polarization ($\mathbf{E} \perp \mathbf{B}$). PL intensity of the same transition as a function of the magnetic field for (b) 3 and (c) 4ML thick nanoplatelets.

even at zero magnetic field [16].

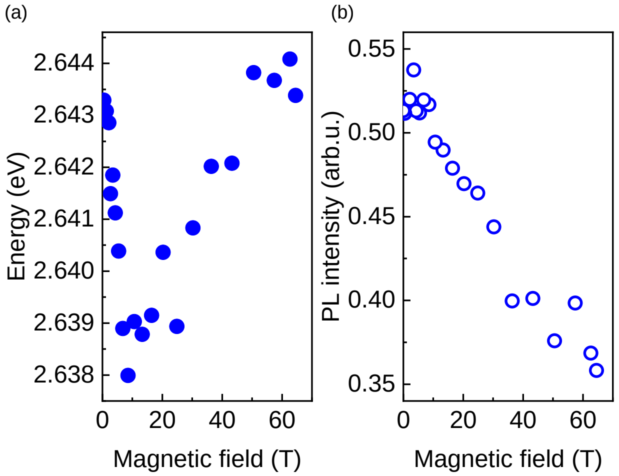


FIG. S8. (a) PL energy and (b) intensity of the 2.64 eV peak of the 3ML thick nanoplatelets measured in transversal polarization as a function of the applied magnetic field.

[1] M. Gramlich, M. W. Swift, C. Lampe, J. L. Lyons, M. Döblinger, A. L. Efros, P. C. Sercel, and A. S. Urban, Dark and bright excitons in halide perovskite nanoplatelets, Advanced Science **9**, 2103013 (2022).

[2] M. Bayer, O. Stern, A. Kuther, and A. Forchel, Spectroscopic study of dark excitons in $In_xGa_{1-x}As$ self-assembled quantum dots by a magnetic-field-induced symmetry breaking, Physical Review B **61**, 7273 (2000).

[3] T. Kataoka, T. Kondo, R. Ito, S. Sasaki, K. Uchida, and N. Miura, Magneto-optical study on excitonic spectra in $(C_6H_{13}NH_3)_2PbI_4$, Physical Review B **47**, 2010 (1993).

[4] M. Baranowski, K. Galkowski, A. Surrente, J. Urban, Ł. Kłopotowski, S. Mackowski, D. K. Maude, R. Ben Aich, K. Boujdaria, M. Chamarro, C. Testelin, P. K. Nayak, M. Dollman, H. J. Snaith, R. J. Nicholas, and P. Plochocka, Giant fine structure splitting of the bright exciton in a bulk $MAPbBr_3$ single crystal, Nano Letters **19**, 7054 (2019).

[5] M. Fu, P. Tamarat, H. Huang, J. Even, A. L. Rogach, and B. Lounis, Neutral and charged exciton fine structure in single lead halide perovskite nanocrystals revealed by magneto-optical spectroscopy, Nano Letters **17**, 2895 (2017).

[6] E. Kirstein, D. Yakovlev, M. Glazov, E. Zhukov, D. Kudlacik, I. Kalitukha, V. Sapega, G. Dimitriev, M. Semina, M. Nestoklon, E. Ivchenko, N. Kopteva, D. Dirin, O. Nazarenko, M. Kovalenko, A. Baumann, J. Höcker, V. Dyakonov, and M. Bayer, The landé factors of electrons and holes in lead halide perovskites: universal dependence on the band gap, arXiv preprint arXiv:2112.15384 (2021).

[7] M. Dyksik, H. Duim, D. K. Maude, M. Baranowski, M. A. Loi, and P. Plochocka, Brightening of dark excitons in 2D perovskites, Science Advances **7**, eabk0904 (2021).

[8] Z. Yu, Effective-mass model and magneto-optical properties in hybrid perovskites, Scientific Reports **6**, 28576 (2016).

[9] M. Snelling, E. Blackwood, C. McDonagh, R. Harley, and C. Foxon, Exciton, heavy-hole, and electron g factors in type-I $GaAs/Al_xGa_{1-x}As$ quantum wells, Physical Review B **45**, 3922 (1992).

[10] B. J. Bohn, Y. Tong, M. Gramlich, M. L. Lai, M. Döblinger, K. Wang, R. L. Hoye, P. Müller-Buschbaum, S. D. Stranks, A. S. Urban, L. Polavarapu, and J. Feldmann, Boosting tunable

blue luminescence of halide perovskite nanoplatelets through postsynthetic surface trap repair, Nano Letters **18**, 5231 (2018).

[11] K. Ema, K. Umeda, M. Toda, C. Yajima, Y. Arai, H. Kunugita, D. Wolverson, and J. Davies, Huge exchange energy and fine structure of excitons in an organic-inorganic quantum well material, Physical Review B **73**, 241310 (2006).

[12] K. Tanaka, T. Takahashi, T. Kondo, K. Umeda, K. Ema, T. Umebayashi, K. Asai, K. Uchida, and N. Miura, Electronic and excitonic structures of inorganic–organic perovskite-type quantum-well crystal $(C_4H_9NH_3)_2PbBr_4$, Japanese Journal of Applied Physics **44**, 5923 (2005).

[13] T. Goto, S. Taguchi, K. Cho, Y. Nagamune, S. Takeyama, and N. Miura, Magneto-optical effect of the wannier exciton in a biaxial $ZnP_2$ crystal. III, Journal of the Physical Society of Japan **59**, 773 (1990).

[14] T. Goto, H. Makino, T. Yao, C. Chia, T. Makino, Y. Segawa, G. A. Mousdis, and G. C. Papavassiliou, Localization of triplet excitons and biexcitons in the two-dimensional semiconductor $(CH_3C_6H_4CH_2NH_3)_2PbBr_4$, Physical Review B **73**, 115206 (2006).

[15] Y. Wang, F. Song, Y. Yuan, J. Dang, X. Xie, S. Sun, S. Yan, Y. Hou, Z. Lou, and X. Xu, Strong triplet-exciton–lo-phonon coupling in two-dimensional layered organic–inorganic hybrid perovskite single crystal microflakes, Journal of Physical Chemistry Letters **12**, 2133 (2021).

[16] W. Choi, S. H. Nam, H.-K. So, S.-E. Lee, M.-H. Jung, and J. I. Jang, Impact of dark excitons on the population and relaxation kinetics of two-dimensional biexcitons in $[CH_3(CH_2)_3NH_3]_2Pb_{1-x}Mn_xBr_4$ ($x = 0 - 0.09$), Journal of the American Chemical Society **143**, 19785 (2021).
